# Varying the item format improved the range of measurement in patient-reported outcome measures assessing physical function

**DOI:** 10.1186/s13075-017-1273-5

**Published:** 2017-03-21

**Authors:** Gregor Liegl, Barbara Gandek, H. Felix Fischer, Jakob B. Bjorner, John E. Ware, Matthias Rose, James F. Fries, Sandra Nolte

**Affiliations:** 1 Department of Psychosomatic Medicine, Center for Internal Medicine and Dermatology, Charité - Universitätsmedizin Berlin, Charitéplatz 1, 10117 Berlin, Germany; 2 Department of Quantitative Health Sciences, University of Massachusetts Medical School, Worcester, MA USA; 3 John Ware Research Group, Watertown, MA USA; 4 Institute for Social Medicine, Epidemiology and Health Economics, Charité – Universitätsmedizin Berlin, Berlin, Germany; 5 National Research Centre for the Working Environment, Copenhagen, Denmark; 6 Optum, Lincoln, RI USA; 7 Department of Public Health, University of Copenhagen, Copenhagen, Denmark; 8 Department of Immunology and Rheumatology, Stanford University School of Medicine, Palo Alto, CA USA; 9 Population Health Strategic Research Centre, School of Health and Social Development, Deakin University, Melbourne, VIC Australia

**Keywords:** Physical function, Patient-reported outcomes, Ceiling effects, Measurement range, Item-response theory, Item information, Response scale, Item format

## Abstract

**Background:**

Physical function (PF) is a core patient-reported outcome domain in clinical trials in rheumatic diseases. Frequently used PF measures have ceiling effects, leading to large sample size requirements and low sensitivity to change. In most of these instruments, the response category that indicates the highest PF level is the statement that one is able to perform a given physical activity without any limitations or difficulty. This study investigates whether using an item format with an extended response scale, allowing respondents to state that the performance of an activity is easy or very easy, increases the range of precise measurement of self-reported PF.

**Methods:**

Three five-item PF short forms were constructed from the Patient-Reported Outcomes Measurement Information System (PROMIS®) wave 1 data. All forms included the same physical activities but varied in item stem and response scale: format A (“Are you able to …”; “without any difficulty”/“unable to do”); format B (“Does your health now limit you …”; “not at all”/“cannot do”); format C (“How difficult is it for you to …”; “very easy”/“impossible”). Each short-form item was answered by 2217–2835 subjects. We evaluated unidimensionality and estimated a graded response model for the 15 short-form items and remaining 119 items of the PROMIS PF bank to compare item and test information for the short forms along the PF continuum. We then used simulated data for five groups with different PF levels to illustrate differences in scoring precision between the short forms using different item formats.

**Results:**

Sufficient unidimensionality of all short-form items and the original PF item bank was supported. Compared to formats A and B, format C increased the range of reliable measurement by about 0.5 standard deviations on the positive side of the PF continuum of the sample, provided more item information, and was more useful in distinguishing known groups with above-average functioning.

**Conclusions:**

Using an item format with an extended response scale is an efficient option to increase the measurement range of self-reported physical function without changing the content of the measure or affecting the latent construct of the instrument.

**Electronic supplementary material:**

The online version of this article (doi:10.1186/s13075-017-1273-5) contains supplementary material, which is available to authorized users.

## Background

Patient-reported outcome (PRO) measures assessing health-related quality of life (HRQoL) have become an essential part of health outcomes research, clinical trials, epidemiological studies, and routine patient monitoring [1–3]. Physical function (PF) is one of the most frequently assessed HRQoL domains [4–6] and has been identified as a core PRO in clinical trials in rheumatic diseases [7]. Thus, efficient assessment of PF is very important. However, traditional PF instruments with a fixed number of items, such as the 10-item Medical Outcome Study Short Form-36 (MOS SF-36®) Health Survey physical functioning scale (PF-10) [8] and the 20-item Health Assessment Questionnaire Disability Index (HAQ-DI) [9], have to compromise between clinical practicality and measurement precision, leading to a limited measurement range on the continuum of physical ability [10].

With the application of item response theory (IRT), any number of items measuring the same latent trait can be calibrated on a common metric. Hence, IRT provides a flexible solution for the challenge of providing practical but still highly precise PRO assessment on a wide range of the latent trait continuum [11–14]. The National Institutes of Health (NIH)-funded Patient-Reported Outcomes Measurement Information System (PROMIS®) has been applying this approach for over 10 years, thereby demonstrating the relevance of IRT item calibration.

PROMIS has developed item banks for a large number of HRQoL domains [2, 15–19], including physical function [10, 20–22]. An important advantage of providing a bank of items scaled on a common metric is that scores derived from different item subsets are directly comparable. This enables the comparison of scores from tailored short forms, which are developed by choosing only the most informative items for a pre-specified trait level and individualized scores from computerized adaptive tests (CATs) [12, 23, 24]. Similarly, if items from different instruments (e.g., short forms) are scaled on the same metric, the measurement precision of these instruments can be directly compared in various populations of interest [25, 26]. This is possible because IRT allows the measurement error of each item (and item subset) to be investigated at each level of the latent trait [27].

Using IRT methods, it has been demonstrated that most PRO instruments measuring PF have satisfactory measurement precision on below average to average functional levels [25, 28]. However, as these instruments have usually been developed for clinical use, they often have ceiling effects in the general population and in samples with higher levels of PF, meaning that a high percentage of these participants achieve the best possible score [29–31]. Thus, individuals with average or above average PF cannot be assessed precisely, leading to low sensitivity to change and larger sample size requirements in clinical trials [28, 29]. The most frequently proposed solution to respond to this shortcoming is the use of items with more difficult content to increase test information on the upper end of a trait continuum [32]. However, this approach might not always be sufficient, e.g., when aiming at extending the measurement range of a static instrument with a fixed number of items or when ceiling effects are still present even after adding new items with more difficult content [33]. In such cases, the modification of the item format of existing items, e.g., by extending the response scale, may present an efficient way of adjusting for ceiling effects [34–36].

Physical function item formats may vary with regard to the item stem, tense (past or present), recall period, attribution (e.g., attribution to health), or response options [4, 35, 37, 38]. For example, in two of the most widely used scales (PF-10, HAQ-DI), the response category that indicates the highest level of PF is the statement that one is able to perform a given activity without any limitations or difficulty [8, 9]. However, there are alternative response scales, for example the one used in the Louisiana State University Health Status Instrument (LSU HSI) [36], that allow respondents to state that the performance of a given activity is easy or even very easy. Such an extended response scale potentially raises the measurement ceiling of PF measures, thus avoiding the necessity of writing new items to measure the ability to perform more difficult activities.

To date, the effect of the item format on item performance in terms of extending the measurement range of PRO measures of PF has not been investigated systematically. To examine the hypothesis that a response format that asks about the ease of doing an activity improves the measurement range, a modification of the LSU HSI item format was incorporated into a set of experimental items in the PROMIS wave 1 data collection [35]. This study uses PROMIS data and IRT to calibrate three five-item short forms with similar content but different item formats on a common metric, to compare the measurement precision and validity of this new item format with two widely used item formats derived from the HAQ-DI and the SF-8™ Health Survey [39].

## Methods

### Development of the PROMIS PF item bank

To establish the PROMIS PF item bank, a stepwise process integrating qualitative and quantitative item identification and evaluation methods was performed [10, 22, 35], following standard PROMIS procedures [19, 40]. The aim was to develop a generic item bank for use in various patient populations to enable the precise assessment of PF, defined as the capability “to carry out activities that require physical actions, ranging from self-care (activities of daily living) to more complex activities that require a combination of skills, often within a social context” [41].

As detailed elsewhere [35], an initial systematic search for PF instruments resulted in the preliminary retention of 168 unique items, which were rewritten to establish a consistent item structure for the PROMIS item bank. This set of 168 revised items was then field tested in the general population and in clinical samples in the USA (total n = 15,817) and analyzed applying established standard criteria for PROMIS item bank development [39]. To minimize the burden on respondents, items were administered in two different designs: (1) a “full bank” design in which separate subsamples answered either 112 (form C) or 56 (form G) PF items and (2) a balanced incomplete “block” design in which subsamples answered blocks of 21 PF items and items for other PROMIS domains. As a result, each PF item was answered by 2201 to 2926 participants [19, 22]. After psychometric evaluation, the final PROMIS PF item bank version 1.0 consisted of 124 items [22].

### Experimental items

Because preparatory analyses showed that the item formats derived from the HAQ-DI [9] (format A: prefaced with “Are you able to …?”; this included five response categories ranging from “without any difficulty” to “unable to do”) and the SF-8 [37] (format B: prefaced with “Does your health now limit you …?”; this included five response options ranging from “not at all” to “cannot do”) revealed appropriate psychometric properties [10] and appeared to be the formats most comprehensible to participants in a pre-test, these two formats were predominantly used for the aforementioned set of 168 items for field testing [35]. However, for experimental reasons, in a small number of items a modified LSU HSI [36] item format was used (format C: prefaced with “How difficult is it for you …”; this included six response options ranging from “very easy” to “impossible”).

To compare the influence of these item formats on item performance, the set of 168 items included 15 experimental items: 5 instrumental activities of daily living (IADLs) of different difficulty levels were presented in all three aforementioned item formats. These three sets of five items differed with regard to the number of response options, definition of the highest and lowest response categories, and attribution to health or not (Table 1). As a result, three five-item short forms with similar content (IADLs) but different item formats were constructed. Of the 15 experimental items, 5 were used in the final 124-item PROMIS PF item bank, with 3 presented in format A and 2 presented in format B.

**Table 1:** Experimental PROMIS PF items for five activities administered in three different item formats

Item format	Item	Item stem	Item content	Number and wording of response options	Attribution to health
A	A1	Are you able to …	… do two hours of physical labor?	5 Without any difficulty 4 With a little difficulty 3 With some difficulty 2 With much difficulty 1 Unable to do	No
	A2^a^		… do yard work like raking leaves, weeding or pushing a lawn mower?		
	A3		… climb several flights of stairs?		
	A4^a^		… go for a walk of at least 15 minutes?		
	A5^a^		… open previously opened jars?		
B	B1^a^	Does your health now limit you in …	… doing two hours of physical labor?	5 Not at all 4 Very little 3 Somewhat 2 Quite a lot 1 Cannot do	Yes
	B2		… doing yard work like raking leaves, weeding or pushing a lawn mower?		
	B3^a^		… climbing several flights of stairs?		
	B4		… going for a walk of at least 15 minutes?		
	B5		… opening previously opened jars?		
C	C1	How difficult is it for you to …	… do two hours of physical labor?	6 Very easy 5 Easy 4 Slightly difficult 3 Difficult 2 Very difficult 1 Impossible	No
	C2		… do yard work like raking leaves, weeding or pushing a lawn mower?		
	C3		… climb several flights of stairs?		
	C4		… go for a walk of at least 15 minutes?		
	C5		… open previously opened jars?		

^a^ Item is part of the final Patient Reported Outcomes Measurement Information System Physical Function (PROMIS PF) item bank version 1.0

### Data analysis

#### Item bank evaluation and calibration

Sufficient unidimensionality of the final 124-item PROMIS PF bank had previously been established [22] and was re-evaluated including the 10 additional experimental items, using confirmatory factor analysis (CFA) of a one-factor model with a weighted least squares means and variance adjusted (WLSMV) estimator and a bifactor model, specifying local factors for items that shared the same response format. CFA analyses of experimental items in format A used data from “full bank” form C (97 items total), while analysis of formats B and C experimental items used data from “full bank” form G (37 items total); for more information on study design, see [22]. A potential problem of local independence between similar items in Format B and C being administered to the same group was evaluated by analyzing residual correlations. Residual correlation of 0.25 or more was considered potentially problematic and the impact on IRT item parameters was evaluated, as previously described [22].

A graded response model (GRM) was fitted to the set of 134 items consisting of the 15 experimental items (three format-specific short forms) and the remaining 119 items of the final PROMIS PF item bank. Due to the data collection design used for the initial set of 168 PF items, some participants answered only a few of the 134 items analyzed in this study. As in previous analyses [22], only participants who responded to at least two of the 134 PF items were included in the GRM. Although GRM item parameters had already been estimated for the 124 items of the final item bank [22], including 5 of the experimental items, the model was re-estimated to include the 10 additional experimental items. As in previous analyses [22], if a specific response category for an item was answered less than three times, the response option was collapsed with the next higher category to ensure stable item parameter estimates. We estimated item parameters comprising item thresholds and item slopes. Threshold parameters define the range on the latent trait continuum at which a particular response is most likely. The slope parameter specifies the discriminative value of an item. Item fit was evaluated using the *S*-*X* ^2^ statistic.

For estimating individual PF scores, we used the expected-a-posteriori method to calculate theta scores that were subsequently linearly transformed to a *T*-metric (mean = 50, SD = 10 in the calibration sample used in this analysis). To determine the precision of a particular item, we calculated item information functions (IIFs), defining the contribution of an item to the overall precision of the item bank at a given *T*-score level [27]. Differences between IIFs resulting from varying the item format were visualized using item information curves (IICs). Using natural cubic spline interpolation, we calculated the area under the curve (AUC) for each IIC on the empirically observed *T*-score range in the calibration sample as a measure of overall item information. To investigate systematic differences in measurement precision depending on the item format used, we first calculated test information functions for each of the format-specific short forms by summarizing respective IIFs and then we compared the resulting format-specific test information curves and related AUCs.

#### Simulation study

Due to the study design, no participant in the calibration sample responded to any of the five IADLs used in the experimental items in all three formats. Therefore, to illustrate the performance of all three formats simultaneously, we used simulated data, following the approach used by Voshaar et al. to evaluate PROMIS PF CATs [25]. In the first step, we simulated “true” PF *T*-scores based on the PF score distributions found for five groups in the calibration sample with different self-reported general health; 10,000 “true” PF *T*-scores were simulated for each of the following five general health groups:

1. Poor general health group: mean PF *T*-score = 35.6 (SD = 6.5)
2. Fair general health group: mean PF *T*-score = 41.9 (SD = 7.6)
3. Good general health group: mean PF *T*-score = 48.9 (SD = 7.8)
4. Very good general health group: mean PF *T*-score = 54.4 (SD = 7.2)
5. Excellent general health group: mean PF *T*-score = 58.8 (SD = 6.5)

In the next step, we simulated responses to the 134 PROMIS PF items for all 50,000 respondents based on their “true” score and the item parameters from the GRM. We scored the three format-specific five-item short forms and the 124-item final PROMIS PF item bank (from now on referred to as the “full bank”) using the simulated responses to the respective items in each of these measures.

To illustrate differences in measurement precision due to item format, we calculated root mean square errors (RMSEs) between simulated true scores and corresponding short form scores, with lower values indicating better agreement in estimating individual PF levels [42].

To illustrate how the differences in item format affect the ability to distinguish groups with different levels of PF, we calculated relative validity (RV) coefficients for each format-specific short form [22, 43]. The RV coefficients were calculated using the analysis of variance (ANOVA) *F*-statistic resulting from comparing the full bank PF scores between general health groups as the denominator and the *F*-statistic from comparing short form PF scores between general health groups as the numerator. Hence, the RV coefficient specifies how well a five-item short form with a specific item format distinguishes among groups that differ in PF, compared to using all 124 items of the original PROMIS PF item bank. We calculated 95% confidence intervals for the RV coefficients using standard bootstrap techniques [43, 44]. To provide RV coefficients for different levels of PF, four different general health group comparisons were performed:

1. Full sample (ANOVA between all five general health groups; n = 50,000)
2. Average PF compilation (ANOVA between groups with fair, good, and very good general health; n = 30,000)
3. Below-average PF compilation (ANOVA between groups with poor general health and fair general health; n = 20,000)
4. Above-average PF compilation (ANOVA between groups with very good and excellent general health; n = 20,000)

CFAs were conducted using Mplus 7.4 [45]. All other statistical analyses were conducted using R 3.1.2 [46]. We used the packages mirt [47] for estimating the GRM and simulating response patterns. For calculating AUCs, we used the package MESS [48]. For plotting item and test information curves, we used ggplot2 [49].

## Results

### Sample

A total of 15,719 subjects responded to at least two of the 134 items analyzed in this study and therefore were included in the GRM. Of these, only 10 subjects (<0.1%) responded to fewer than 6 items; 99.7% responded to at least 12 items. More than half (54%; n = 8568) responded to one or more of the 15 experimental items (sample characteristics in Additional file 1: Table S1). The experimental items were answered by 2217–2835 participants. The calibration sample had a wide range of PF, with empirically observed *T*-scores (mean = 50, SD = 10) ranging from 11.1 to 73.6.

### Evaluation of unidimensionality

Form C and form G had satisfactory fit for the one-factor solution. Factor loadings for the experimental items ranged between 0.83 and 0.93 (format A), 0.83 and 0.96 (format B), and 0.72 and 0.92 (format C). We found residual correlation above 0.25 in one only pair of items (B5 and C5, *r* = 0.30). However, excluding item B5 in the GRM calibration did not notably affect the parameters of item C5 and vice versa, so both items were retained. In the bifactor models, loadings on the global PF factor were substantially higher than loadings on local factors defined by the common response format, thus supporting sufficient unidimensionality of the experimental items and the original PF item bank. For more details, see Additional file 2: Table S2.

### Item properties

The results of the IRT analyses for the 15 experimental items (5 IADLs presented in three different item formats) are summarized in Table 2. When adjusting for multiple testing, no item fit-statistic showed significant misfit for any experimental item. Except for one IADL (“open previously opened jars”), item slopes were generally high for all formats. Items prefaced with “Does your health now limit you …” (format B) tended to show slightly higher slope parameters compared to formats A and C (see Table 2).

**Table 2:** Psychometric results for the experimental items presented in three different item formats

Item	Format^b^	Content	Slope	Threshold^c^						Item fit: *p* (*S*-*X* ^2^)^d^	I_max_ (at *T*-score)^e^	Area under the curve^f^	Percentage floor/percentage ceiling^g^
				1	2	3	4	5	Mean				
A1	A	Do 2 hours of physical labor	3.49	38.6	42.9	47.8	54.9		46.1	0.6523	3.71 (T = 42)	92.9	10.4/41.6
B1^a^	B		4.53	38.0	43.0	48.4	53.1		45.6	0.1133	5.93 (T = 49)	132.9	10.0/42.7
C1	C		4.01	37.7	42.0	46.3	52.7	59.8	47.7	0.0358	4.88 (T = 42)	140.3	10.2/19.6
A2^a^	A	Do yard work	4.09	36.3	40.1	44.3	50.7		42.9	0.1473	5.10 (T = 40)	111.1	6.6/57.3
B2	B		4.79	35.7	40.8	46.1	50.6		43.3	0.0751	6.58 (T = 47)	144.0	6.7/52.7
C2	C		4.53	34.3	39.1	43.1	49.3	56.0	44.4	0.0300	6.10 (T = 40)	167.5	5.3/32.1
A3	A	Climb several flights of stairs	3.78	35.2	40.3	45.2	52.0		43.2	0.1722	4.28 (T = 41)	107.0	5.8/51.5
B3^a^	B		4.20	34.2	40.8	46.7	51.3		43.3	0.8460	5.16 (T = 48)	126.0	5.1/51.3
C3	C		3.78	33.3	39.8	44.0	51.0	57.1	45.0	0.1174	4.31 (T = 42)	135.0	6.3/25.4
A4^a^	A	Go for a walk of at least 15 minutes	3.78	33.2	36.4	40.2	45.5		38.8	0.2497	4.45 (T = 37)	91.3	3.7/73.5
B4	B		4.03	32.1	37.2	42.0	45.8		39.3	0.3555	4.93 (T = 43)	107.0	3.4/71.6
C4	C		3.99	30.3	35.6	39.5	44.9	50.8	40.2	0.0033	4.85 (T = 37)	134.7	3.6/47.5
A5^a^	A	Open previously opened jars	1.91	18.8	28.4	37.9			28.4	0.2434	1.10 (T = 28)	36.5	0.9/85.8
B5	B		1.90	12.9	22.8	32.3	39.6		26.9	0.5429	1.10 (T = 33)	39.9	0.3/81.9
C5	C		1.57	5.0	15.5	23.4	34.0	45.4	24.7	0.1877	0.77 (T = 20)	33.6	0.3/62.4

^a^Item is part of the final Patient Reported Outcomes Measurement Information System Physical Function (PROMIS PF) item bank version 1.0. ^b^Format A: “Are you able to …” (five-category response scale from “Without any difficulty” to “Unable to do”); format B: “Does your health now limit you in …” (five-category response scale from “Not at all” to “Cannot do”); format C: “How difficult is it for you to …” (six-category response scale from “Very easy” to “Impossible”). ^c^Thresholds are transformed to a *T*-score of 50 ± 10, where 50 = mean and 10 = standard deviation of the analytic sample; slopes are reported unchanged. ^d^ *X* ^2^ statistics (*S*-*X* ^2^) were evaluated after adjusting for multiple testing (*p* < 0.0033). ^e^I_max_ (at *T*-score) depicts the maximum of item information (upper number) of a given item at the corresponding point on the *T*-score continuum. ^f^Total area under the item information curve (IIC) on the empirically observed *T*-score range in the calibration sample (*T*-score = 11.1–73.6). ^g^Percentage of participants who answered the item with the lowest (floor) or highest (ceiling) possible response category

Item thresholds tended to be similar for format A and format B. In contrast, using format C with the item stem “How difficult is it for you to …” and an extended six-category response scale (ranging from “impossible” to “very easy”) expanded the range of the thresholds on the latent trait continuum in both directions. This was particularly pronounced at the positive end of the continuum where the last response in format C increased the measurement range by ≥0.5 SDs of the PF distribution of the sample for all physical activities. As a consequence, the percentage of participants who responded with the highest possible response category was systematically lower (by about 20–25% of the total sample) for items presented in format C compared to the other formats. For two of the more difficult activities (2 hours of physical labor and climbing several flights of stairs), the ceiling effects were halved when using format C compared to both format A and format B (see Table 2).

**Fig. 1:**
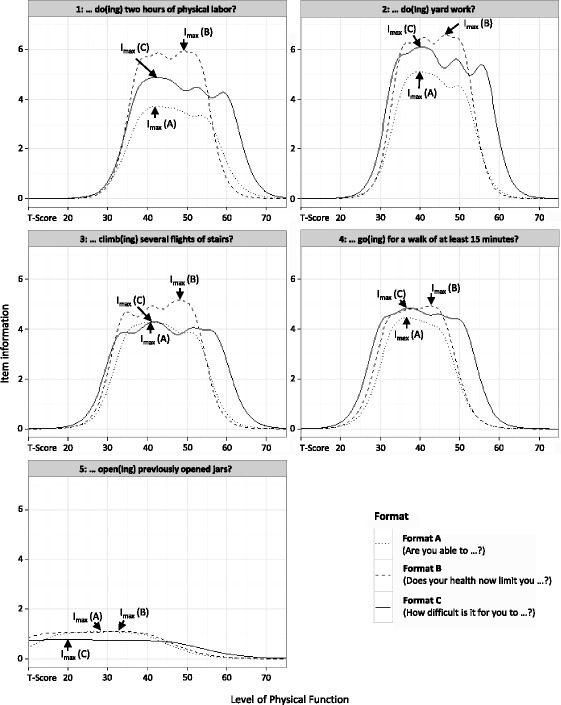
Comparison of item information functions (IIFs) using different item formats. Format A: “Are you able to …” (five-category response scale from “Without any difficulty” to “Unable to do”); format B: “Does your health now limit you in …” (five-category response scale from “Not at all” to “Cannot do”); format C: “How difficult is it for you to …” (six-category response scale from “Very easy” to “Impossible”). Item parameters and IIFs were initially estimated using a standard normal physical function (PF) metric. PF values were subsequently transformed to a *T*-metric, where 50 = mean and 10 = standard deviations of the analytic sample (*x-axis*). Item information values on the *y-axis* are reported unchanged. *I*_*max*_ depicts the specific point on the *T*-score continuum, where a given item delivers maximum item information

Figure 1 depicts the IICs for all experimental items presented in different item formats. Format B delivered the highest maximum item information for four of the five physical activities. Moreover, the maximum item information of format B was placed on a systematically higher point on the PF continuum compared to the other formats. In contrast, format C had the broadest measurement range on the *T*-score continuum for each of the five physical activities. The maximum item information of a given item and corresponding points on the latent trait and the AUCs are presented in Table 2. The highest overall item information as specified by the AUC was found for format C except for items asking about opening previously opened jars.

**Fig. 2:**
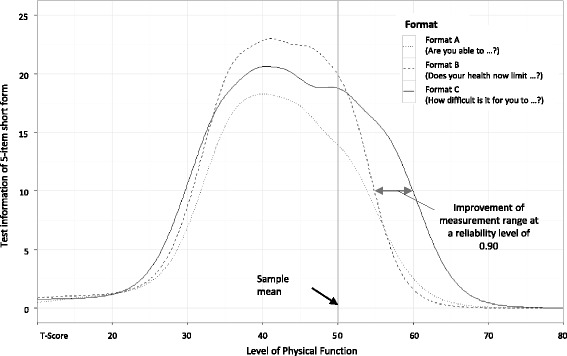
Comparison of test information functions between different item formats. Format A: “Are you able to …” (five-category response scale from “Without any difficulty” to “Unable to do”); format B: “Does your health now limit you in …” (five-category response scale from “Not at all” to “Cannot do”); format C: “How difficult is it for you to …” (six-category response scale from “Very easy” to “Impossible”). Item parameters and information functions were initially estimated using a standard normal physical function (PF) metric. PF values were subsequently transformed to a *T*-metric, where 50 = mean and 10 = standard deviations of the analytic sample (*x-axis*). Test information values on the *y-axis* are reported unchanged

Consequently, the item format affected the total test information provided by the short forms (Fig. 2). The highest maximum test information was found for format B, while items with an extended response format (format C) were highly informative on the widest range on the latent continuum. That is, format C increased the range of highly reliable measurements (defined as marginal reliability ≥0.9 ≈ test information ≥10) by about 0.5 SDs of the PF distribution of the sample on the positive side of the continuum and about 0.1 to 0.2 SDs on the negative side of the continuum.

The cumulative AUC for format C (AUC = 611) was 39% larger than for format A (AUC = 439) and 11% larger than for format B (AUC = 550). When focusing on the item information curve for *T*-scores above 50, the cumulative AUC for Format C (AUC = 192) was 109% larger than for format A (AUC = 92) and 81% larger than for format B (AUC = 106).

### Agreement between true scores and short forms

The results of the simulation study indicated that the agreement between the simulated true scores and the estimated short form scores was generally lower for formats A and B than for format C (Table 3). Using formats A and B, the agreement with the simulated true scores became even lower when analyzing groups with average to high PF levels (up to RMSE of 4.3 for format A and RMSE of 4.4 for format B). In contrast, the agreement between simulated true scores and short form scores remained relatively constant among all groups when using format C, even in individuals with excellent general health (RMSE ≤3.3).

**Table 3:** PROMIS PF full bank and short form scoring characteristics and agreement with simulated “true” scores

General health groups	True PF *T*-score^a^ mean (SD)	Full bank (124 items)^b^			Format A^e^ (5-item short form)			Format B (5-item short form)			Format C (5-item short form)		
		*T*-score mean (SD)	RMSE^c^	Percentage floor/percentage ceiling^d^	*T*-score mean (SD)	RMSE	Percentage floor/percentage ceiling	*T*-score mean (SD)	RMSE	Percentage floor/percentage ceiling	*T*-score mean (SD)	RMSE	Percentage floor/percentage ceiling
Poor	35.6 (6.5)	35.7 (6.4)	0.7	0.0/0.0	36.6 (6.3)	3.0	3.9/0.2	36.4 (6.3)	2.7	1.5/0.2	36.3 (6.3)	2.7	0.5/0.0
Fair	41.9 (7.6)	41.9 (7.7)	0.8	0.0/0.0	42.5 (7.9)	2.9	1.3/3.8	42.3 (790)	2.6	0.5/4.6	42.3 (7.7)	2.5	0.2/0.7
Good	48.9 (7.8)	49.0 (7.9)	1.1	0.0/0.1	49.4 (8.0)	3.2	0.1/17.0	49.4 (8.0)	3.1	0.0/21.5	49.1 (7.9)	2.6	0.0/6.2
Very good	54.4 (7.2)	54.5 (7.3)	1.5	0.0/0.2	54.5 (6.9)	3.8	0.0/37.9	54.5 (6.8)	3.7	0.0/45.4	54.4 (7.3)	2.9	0.0/16.8
Excellent	58.8 (6.5)	58.7 (6.4)	1.9	0.0/0.7	57.8 (5.3)	4.3	0.0/59.0	57.8 (5.0)	4.4	0.0/67.1	58.4 (6.3)	3.3	0.0/32.6
Full sample	47.9 (11.0)	48.0 (11.0)	1.3	0.0/0.2	48.1 (10.4)	3.5	1.1/23.6	48.1 (10.5)	3.4	0.4/27.8	48.1 (10.5)	2.8	0.1/11.3

^a^ *T*-scores have a mean of 50 and standard deviation of 10 in the analytic sample. ^b^Final Patient Reported Outcomes Measurement Information System Physical Function (PROMIS PF) item bank version 1.0. ^c^RSME = root mean square error between estimated *T*-scores and simulated “true” *T*-scores. ^d^Percentage of the simulated sample who reached the lowest (“floor”) or highest (“ceiling”) possible score. ^e^Format A: “Are you able to …” (five-category response scale from “Without any difficulty” to “Unable to do”); Format B: “Does your health now limit you in …” (five-category response scale from “Not at all” to “Cannot do”); format C: “How difficult is it for you to…” (six-category response scale from “Very easy” to “Impossible”)

The highest possible short form *T*-score was 61.8 when using format A and 61.0 when using format B. In contrast, format C allowed for *T*-scores up to 65.5, which reduced ceiling effects by more than half in the full simulated sample. Format C was found to be especially beneficial for groups with high PF levels. For example, in the subgroup with “very good” general health, 45.4% of the simulated sample reached the highest possible short form score when using format B. In contrast, only 16.8% of the subgroup with “very good” health reached the highest possible score when using format C. Moreover, lower floor effects were found when using format C.

### Distinguishing known groups

The results of the RV analyses using simulated data are presented in Table 4. In most group comparisons (comparisons a, b, and c) the RV was 0.90 or above for all item formats. In contrast, when distinguishing between the two groups with “very good” and “excellent” general health (comparison d), the RV coefficients of format A (RV = 0.79; 95% CI = 0.74–0.84) and format B (RV = 0.78; 95% CI = 0.74–0.83) were considerably lower compared to format C (RV = 0.92; 95% CI = 0.88–0.96).

**Table 4:** Analysis of variance (ANOVA) and relative validity (RV)

Subgroup comparisons	General health groups considered for ANOVA^a^					Full bank (124 items)^b^		Format A^c^ (5-item short form)		Format B (5-item short form)		Format C (5-item short form)	
	Poor	Fair	Good	Very good	Excellent	F	RV	F	RV^d^ (95% CI)	F	RV (95% CI)	F	RV (95% CI)
a. Full sample	X	X	X	X	X	16,957	1 .0	15,582	0.92 (0.91–0.93)	16,139	0.95 (0.94–0.96)	15,712	0.93 (0.92–0.94)
b. Average PF		X	X	X		6960	1 .0	6246	0.90 (0.88–0.91)	6473	0.93 (0.92–0.94)	6349	0.91 (0.90–0.93)
c. Below-average PF	X	X				3818	1 .0	3421	0.90 (0.87–0.92)	3491	0.91 (0.89–0.94)	3564	0.93 (0.91–0.96)
d. Above-average PF				X	X	1870	1 .0	1476	0.79 (0.74–0.84)	1467	0.78 (0.74–0.83)	1720	0.92 (0.88–0.96)

^a^Subgroups marked X were considered for calculating *F* values (ANOVA); n = 10,000 per subgroup. ^b^Final Patient Reported Outcomes Measurement Information System Physical Function (PROMIS PF) item bank version 1.0. ^c^Format A: “Are you able to …” (five-category response scale from “Without any difficulty” to “Unable to do”); format B: “Does your health now limit you in …” (five-category response scale from “Not at all” to “Cannot do”); format C: “How difficult is it for you to …” (six-category response scale from “Very easy” to “Impossible”). ^d^RV calculation: (ANOVA *F* values derived from using a format-specific 5-item short form)/(ANOVA *F* values derived from using full bank scores)

## Discussion

In this study we compared the performance of three different item formats for measuring self-reported PF by analyzing item information. Using simulated data, we illustrated precision in estimating scores and validity in distinguishing between known groups of three five-item short forms with identical content but different item stems and response scales. The five physical activities included in these short forms covered a broad range of item difficulty. Using IRT methodology for data analysis offered the unique opportunity to investigate and visualize measurement precision and range at the item level.

We found strong evidence that the item format may affect the measurement properties of patient-reported PF outcomes. These findings are of practical importance both to researchers and clinicians because this is not only relevant for the development of new instruments but also for the selection of currently available questionnaires for assessing PF in a given population of interest. Moreover, these findings deliver useful information for data interpretation, as the distribution of presumably similar samples can be impacted by the way items are phrased, i.e., identical content but different stem and response format.

In detail, we found that item information differed systematically between the three formats. Format C (“How difficult is it for you to …”), which used an extended response scale including a sixth response option (“very easy”), improved the measurement range by about half a standard deviation on the positive side of the continuum and by about a tenth to a fifth of a standard deviation at the negative end of the continuum, compared to format A (“Are you able to …”) and format B (“Does your health now limit you …”). This finding was consistent across different item difficulties. The improvement of the measurement range was found to be particularly beneficial for groups with above-average PF levels, reducing the number of subjects demonstrating ceiling effects in a five-item short form by half or even more, when using format C instead of the other item formats. As a consequence, format C was the only item format that had relatively constant measurement precision for all PF levels investigated in the simulation study and had sufficient power to distinguish between groups with above-average functioning. As the improved measurement range of format C was particularly apparent at the positive end of the PF continuum, it seems likely that this improvement was not solely caused by using six instead of five response options but rather by allowing subjects to state that activities were “very easy”.

Moreover, our results support that all included item formats measured the same latent construct of PF. The majority of factor loadings were high and their respective magnitude seemed to depend mainly on item content. Consequently, although the final PROMIS PF item bank includes item formats with five-category response options only [35], this study provides evidence that an extended response scale can be applied without affecting the underlying PF construct.

These findings have practical implications for the challenge when encountering ceiling effects, for example, when measuring PF in the general population or in other samples with high PF. The usual way to minimize such ceiling effects is to provide new items with item content that is more relevant for individuals with high PF [32, 33]. However, although providing a larger number of items assessing the extremes of a given trait is undoubtedly useful for the improvement of CATs, this approach does not seem beneficial for increasing the measurement performance of static measures that use the same items for all respondents. Such static measures may still be preferred by many researchers and clinicians for practical reasons [4]. Our findings suggest that it is possible to reduce ceiling effects by optimizing the item format without changing the content of the measures, which may be especially relevant for the future development of items for static PF measures for use in heterogeneous populations with a broad range of ability. However, such modified items should be evaluated psychometrically before use, and additional qualitative item review may be needed. Doing so was beyond the scope of this study.

Another finding of our study is that compared to item formats that do not use attribution, items prefaced with a health-related item stem, as used in format B, delivered the highest maximum item information on a rather narrow range on the PF continuum. Therefore, those types of items seem to be particularly interesting for CATs where highly informative items are selected automatically based on the individual patient’s trait level. Moreover, using format B resulted in increased power to distinguish between known groups with close-to-average PF levels compared to the other formats. However, it is not entirely clear if these benefits of format B are caused by health attribution; another reason could be that the wording in format B focuses on “limitations” while both format A and format C ask for “difficulty” in performing physical activities. Further, slightly lower floor effects were found for format B (using “cannot do” as the lowest response option) than for format A (using “unable to do” as the lowest response option).

Our study has some limitations. First, our conclusions are based on only five items. Consequently, we cannot be sure that our results apply to all items in the PROMIS PF item bank. However, the format-specific differences were highly consistent among all experimental items. A second limitation concerns the selection of only three item formats. Among PRO instruments for the assessment of PF there is a large variety of item formats, which differ in many more aspects than the response scale and item stem [35, 37, 38]. Future studies should clarify whether other formats should be considered for further optimization of measurement precision, and also if the wording of the formats used in this study can be further improved [50]. In particular, modifications might be made to format C, which is based on the LSU HSI (format C: “How difficult is it for you to …”), in which the item stem asked about difficulty but not ease, whereas the corresponding response set included “easy” and “very easy”.

Third, we had to use simulated data for illustrating differences in measurement precision due to the item formats because the study design did not permit direct comparisons using real data. Fourth, it has been shown that PF measures are not only limited by ceiling effects but also by floor effects when assessing highly disabled populations [33]. It seems unlikely that this issue can be solved sufficiently by simply modifying the response scale, as the most extreme response option at the negative end of the trait continuum is usually rated “impossible”. For highly disabled samples, it may therefore be necessary to include items asking about basic activities of daily living (ADLs). Finally, although we found differences in measurement precision between the item formats, it remains unclear whether one of the formats used in this study is superior to the others in measuring what a person is actually able to perform, i.e., as measured by performance-based outcome measures.

## Conclusions

This study systematically investigated differences in measurement properties resulting from extending the response scale of PRO measures assessing PF. Our findings provide evidence that using an extended six-category response format, including the response options “easy” and “very easy”, is an efficient and valid way to considerably extend the range of precise measurement of PF at the positive end of the trait continuum without changing the content of the measure or affecting the latent construct of the instrument. Optimizing the item format offers an effective opportunity to improve measurement precision and to reduce ceiling effects. This is especially relevant for the application of generic short forms in populations with average and above-average levels of PF and for the selection of global items measuring PF.

## Additional files

Additional file 1: Table S1. Summary of sample characteristics. (DOCX 38 kb)
Additional file 2: Table S2. Results of confirmatory factor analyses. (DOCX 38 kb)

## Abbreviations

ADL: Basic activities of daily living
ANOVA: Analysis of variance
AUC: Area under the curve
CAT: Computerized adaptive testing
CFA: Confirmatory factor analysis
GRM: Graded response model
HAQ-DI: Health Assessment Questionnaire Disability Index
HRQoL: Health-related quality of life
IADL: Instrumental activities of daily living
IIC: Item information curve
IIF: Item information function
IRT: Item response theory
LSU HSI: Louisiana State University Health Status Instrument
MOS SF-36: Medical Outcome Study Short Form-36
PF: Physical function
PF-10: Medical Outcome Study Short Form-36 Health Survey Physical Function scale
PRO: Patient-reported outcome
PROMIS: Patient-reported outcomes measurement information system
RMSE: Root mean square error
RV: Relative validity
