# Supplementary material for: Varying the item format improved the range of measurement in patient-reported outcome measures assessing physical function
Source: Arthritis Res Ther. 2017 Mar 21;19:66. doi: 10.1186/s13075-017-1273-5 (PMC5359818; doi:10.1186/s13075-017-1273-5)
Supplement: Additional file 1: Table S1. — Summary of sample characteristics. (DOCX 38 kb) [file 13075_2017_1273_MOESM1_ESM.docx]

| Additional file 1: Table S1. Summary of sample characteristics | | |
| --- | --- | --- |
|  | **Full sample** ^a^ | **Experimental item subsample** ^b^ |
| n | 15,719 | 8,568 |
| Mean age (SD) | 53.85 (16.6) | 54.66 (16.8) |
| % Female | 52.1 | 49.5 |
| % White | 82.7 | 82.5 |
| % > High School graduate | 82.1 | 82.0 |
| % Any chronic condition ^c^ | 77.6 | 76.4 |
| % Musculoskeletal ^d^ | 35.5 | 29.7 |
| % Cardiopulmonary ^d^ | 33.5 | 34.0 |
| % Mental disorder ^d^ | 33.5 | 26.0 |
| % Cancer ^d^ | 19.7 | 27.1 |
| % Gastroenterological ^d^ | 17.7 | 17.6 |
| % Neurological ^d^ | 5.9 | 2.3 |

^a^ All participants who responded to at least two of the 134 items analyzed in the present study

^b^ Subsample of participants who responded to at least one of the experimental items presented in different formats

^c^ Participants were asked for current or previous chronic conditions: ‘Have you ever been told by a doctor or a health professional that you have …’

^d^ Participants who reported musculoskeletal disorders (osteoarthritis (OA), rheumatoid arthritis (RA)), cardiopulmonary conditions (asthma, chronic obstructive pulmonary disease (COPD), coronary heart disease, myocardial infraction, heart failure), mental disorders (depression, anxiety, addiction), cancer of any origin, gastroenterological disorders (diabetes, liver disease), or neurological disorders (amyotrophic lateral sclerosis, multiple sclerosis, spinal cord injury (SCI), Parkinson’s disease). Percentages sum to over 100% as most participants reported more than one condition.
