# Supplementary material for: Varying the item format improved the range of measurement in patient-reported outcome measures assessing physical function
Source: Arthritis Res Ther. 2017 Mar 21;19:66. doi: 10.1186/s13075-017-1273-5 (PMC5359818; doi:10.1186/s13075-017-1273-5)
Supplement: Additional file 2: Table S2. — Results of confirmatory factor analyses. (DOCX 38 kb) [file 13075_2017_1273_MOESM2_ESM.docx]

| Additional file 2: Table S2. Results of Confirmatory Factor Analyses | | | | | |
| --- | --- | --- | --- | --- | --- |
|  | **One-factor model** | |  | **Bifactor model** | |
|  | **Form C ^a^**  *n = 639* | **Form G ^b^**  *n = 757* |  | **Form C ^a^**  *n = 639* | **Form G ^b^**  *n = 757* |
| Chi-square | 7491 (df = 4559),  p < 0.001 | 3869 (df = 629),  p < 0.001 |  | 6182 (df = 4462),  p < 0.001 | 3017 (df = 592),  p < 0.001 |
| CFI | 0.982 | 0.970 |  | 0.989 | 0.977 |
| TLI | 0.982 | 0.968 |  | 0.989 | 0.975 |
| RMSEA | 0.032  [0.030 - 0.33] | 0.083  [0.080 - 0.085] |  | 0.025  [0.023 - 0.026] | 0.074  [0.071 - 0.076] |

RMSEA: Root Mean Square Error of Approximation; CFI: Comparative Fit Index; TLI: Tucker Lewis Index

^a^ Set of 97 items consisting of 5 experimental items using Format A: ‘Are you able to…’ (Five-category response scale from ‘Without any difficulty’ to ‘Unable to do’) and 92 other items of the final PROMIS PF item bank; n=639 subjects responded to all of these items

^b^ Set of 37 items consisting of 10 experimental items using Format B: ‘Does your health now limit you in…’ (Five-category response scale from ‘Not at all’ to ‘Cannot do’) and Format C: ‘How difficult is it for you to…’ (Six-category response scale from ‘Very easy’ to ‘Impossible’) and 27 other items of the final PROMIS PF item bank; n=757 subjects responded to all of these items.
